# Concordance between two intrapersonal psychological resilience scales: how should we be measuring resilience?

**DOI:** 10.1186/s13034-022-00472-z

**Published:** 2022-05-16

**Authors:** Andrés C. Cardozo, Daniel E. Suárez, Lorena A. Bejarano, Elena M. Trujillo, Oscar A. Bernal, Anna E. Ordóñez

**Affiliations:** 1 Research Group on Public Health, Education, and Professionalism, School of Medicine, Universidad de los Andes, Cra 1 Nº 18A - 12, 111711 Bogotá, Colombia; 2 Research Group on Public Health, Education, and Professionalism, Universidad de los Andes, Bogotá, Colombia; 3 Research Group on Public Health, Education, and Professionalism, Alberto Lleras Camargo School of Government, Universidad de los Andes, Bogotá, Colombia; 4 Office of Clinical Research, National Institute of Mental Health, Bethesda, MD USA

**Keywords:** Psychological resilience, Adolescents, School health services, Surveys and questionnaires

## Abstract

**Background:**

While resilience has generated a lot of interest in mental health, operationalizing the construct of resilience remains an important challenge. This study aims to evaluate the concordance of two resilience scales that evaluate intrapersonal aspects of resilience in adolescents.

**Methods:**

Cross-sectional evaluation of internal consistency, concordance, and correlation of the Individual Protective Factors Index Questionnaire (IPFI) and the Adolescent Resilience Scale (ARS) in sixth grade students of three low-income public schools in Colombia.

**Results:**

325 adolescents (41.5% female) participated in the study (72.5% response rate). Mean age was 12.1 years (standard deviation [SD]: 1.04). Of a possible score from 1–4, the mean adjusted IPFI score was 3.3 (SD: 0.3; Cronbach’s alpha: 0.87). Of a possible score from 21–105, the total ARS score was 76.4 (SD 13.0; Cronbach’s alpha: 0.82); both distributions were non-normal and left-skewed. The Lin’s concordance correlation coefficient was 0.34 and the Spearman correlation coefficient was 0.52 (p-value < 0.0001 for both). Notably, 10 adolescents (3.1% of the sample) had a score in the lowest quartile in one of the two instruments, and a score in the highest quartile in the other instrument.

**Conclusions:**

There was low concordance between the scales, with notable lack of overlap in who was identified as having “low” levels of resilience. To better elucidate and operationalize the construct of resilience, studies using resilience scales should consider greater focus in understanding what aspects of the construct are being measured and how they relate to meaningful variables (well-being, risk of illness, etc.).

## Background

Psychological resilience is a theoretical construct that has generated increasing interest in recent years and has been associated with multiple advantageous outcomes [1–5]. In a seminal paper in 1997, Polk postulated that four primary clusters of traits or factors comprised resilience, including dispositional, relational, situational, and philosophical [5]. In the last two decades, research on resilience has been directed to aspects such as systems theory [6], cultural context and associated neurobiological findings, including the individual/internal factors as well as the environmental/external factors of the construct [3, 6]. According to Hjemdal et al., resilience can be divided into essentially three groups: (1) personal characteristics or disposition (dispositional), (2) family cohesion (relational), and (3) social resources outside of the family (situational) [7]. Researchers who work on this framework of intrapersonal (biological and psychological) and interpersonal (environmental and systemic) factors of resilience argue that it is not a fixed personality trait, but a dynamic construct [4]. Resilience can be simply defined as recovering from or positively adapting to illnesses or adversities [3]; however, considering all other aspects, it is also defined as the ability of a dynamic system to adapt satisfactorily to disturbances that threaten its functioning, viability or development [6]. Even so, resilience has also been depicted under other terms, such as hardiness, benefit finding, thriving and posttraumatic growth [8]. Other concepts that are at times referred to as resilience include protective factors, psychological adaptation, and coping [8, 9].

Despite its conceptual variability, resilience has gained so much popularity that it has been increasingly included in health policies [10]. Aligned with this, in Colombia the Department of Health and Social Protection included achieving a 10% increase in resilience levels as part of its Ten-Year Public Health Plan (*Plan Decenal de Salud Pública,* in Spanish) for 2012–2021 [11]. Such policy recommendations are controversial, and some authors believe that there is insufficient evidence for using resilience in the development of public policies and prevention/treatment programs [10, 12]. The dearth of studies on normative data of resilience levels for the general population highlights the lack of emprirical evidence in this field. To date the few population studies that included the assessment of resilience, while notably using different scales, all indicate a seemingly non-normal left-skewed distribution of resilience [13–15]. Such distributions indicate that while the majority of the population studied had high levels of resilience, a small number reported notably low ones. To this point, in addition to more population level data, greater clarity on the implications of lower reported levels of resilience would be helpful to better understand the construct.

These challenges in operationalization of resilience have led to diverse measurement approaches such as qualitative instruments, quantitative scales and measurements of outcomes that try to qualify or quantify a more positive than expected outcome for the severity of various adverse circumstances [9]. The instruments used in quantitative approaches are highly varied. In fact, a systematic review by Pangallo et al. underscores the lack of consensus “as to which dimensions best represent resilience” in adult measures, and considers that future research would benefit from using dimension-specific measures instead of global resilience measures [16]. Another review that includes studies of all age groups also concludes that it is not clear how to choose the best way to measure resilience [17]. These aspects related to operationalizing the concept are particularly important when evaluating interventions designed to improve resilience. [18].

To better understand which resilience measures may be best suited for studies on increasing psychological resilience, greater understanding of how measures of resilience differ and the extent to which a measurement tool can impact the identification of at-risk groups is needed. Comparing resilience data from different instruments can help to understand whether they are measuring the same construct and can identify individuals at risk with similar accuracy. Framed by the public health goal of increasing resilience levels in the general population in Colombia, this study aims to evaluate the concordance in resilience levels measured with two different scales of intrapersonal resilience, previously studied in Spanish-speaking adolescents: The *Individual Protective Factors Index Questionnaire* (IPFI) [19] and the *Adolescent Resilience Scale* (ARS) [20] in a sample of sixth-grade students from three low-income schools in Bogotá, Colombia.

## Methods

We conducted a concordance analysis of two different intrapersonal psychological resilience assessment scales, previously validated in Spanish-speaking adolescents. These data were collected as part of the baseline information for an intervention study aimed at increasing psychological resilience. The study was approved by the Institutional Review Board at *Universidad de los Andes* in Bogotá, Colombia*.*

### Participants

Participants were sixth-grade students of two charter schools and one public school in low-income neighborhoods, geographically close to each other, with similar socio-economic characteristics in Colombia’s capital city, Bogotá. The two charter schools were selected by convenience because they have a long-standing relationship with the University as it has been involved in supporting their educational mission. The public school was selected because of its geographic proximity to the charter schools, leading to similar socio-demographic characteristics. The study team invited all sixth graders and their parents in the three schools to participate in the study by attending parent-teacher conferences to present the study and discuss questions, as well as through the provision of written information about the study via handouts. Consent from parents or legal guardians and assent from adolescents was obtained for all participants. There were no exclusion criteria. Considering that a correlation of 0.2 would be the minimum relevant correlation between any two measures, we calculated that we would need a sample size of N = 194 to detect a correlation of at least 0.2 with a power of 80%.

### Instruments

Self-reported instruments were used through an anonymized online survey. Participants answered the questionnaires on school computers supervised by a school staff member and a member of the research team to ensure participant confidentiality and address any questions regarding the survey. In addition to the resilience measurement tools, questions adapted from Colombian national surveys were included to evaluate demographic characteristics, family structure and functioning, economic indicators, health indicators, indicators of academic performance and spirituality. The measurement of individual resilience levels was carried out using the two instruments at study, selected because they have been validated in Spanish-speaking adolescent populations and intend to measure intrapersonal factors of resilience as described below:

- The Individual Protective Factors Index Questionnaire (IPFI) [19], Spanish version: This scale has 61 items distributed in three domains (social bonding, personal competencies, and social competencies) and 10 dimensions (see Table 1 for the complete list), and each item is scored between 1 and 4. The total score ranges from 61–244. The adjusted score ranges between1-4 and is obtained by dividing the total score by the number of items. It has been validated with good internal consistency for the total scale and fair internal consistency for its dimensions and was designed to measure “*protective factors* associated with healthy personal and social development among youth in high-risk environments” [19].
- The Adolescent Resilience Scale (ARS) [20], validated in Colombia by Quiceno et al. [21]: This scale has 21 items distributed in three factors (novelty seeking, emotional regulation and positive future orientation), and each item is scored between 1 and 5 (total score 21–105 points). It has been validated with good internal consistency results and was designed to measure “psychological features of resilient individuals” [20, 21].

**Table 1:** Outcomes of the Resilience Scales and Subscales Used (N = 325)

	Mean (SD)	Items	Cronbach's alpha	P-value in the normality test*
IPFI adjusted score^	3.3 (0.3)	61	0.87	< 0.01
IPFI—Social bonding—School	20.9 (2.4)	6	0.38	< 0.01
IPFI—Social bonding—Family	18.9 (2.5)	6	0.49	< 0.01
IPFI—Social bonding—Prosocial norms	19.6 (2.7)	6	0.53	< 0.01
IPFI—Personal competencies–Self-concept	20.5 (2.6)	6	0.58	< 0.01
IPFI—Personal competencies–Self-control	18.4 (3.5)	6	0.68	< 0.01
IPFI—Personal competencies–Self-efficacy	16.3 (2.5)	7	0.21	**0.07**
IPFI—Personal competencies–Positive outlook	25.2 (2.4)	6	0.47	< 0.01
IPFI—Social competencies—Assertiveness	18.1 (2.8)	6	0.35	0.68
IPFI—Social competencies—Confidence	19.8 (3.0)	6	0.62	< 0.01
IPFI—Social competencies—Cooperation	21.6 (2.2)	6	0.69	< 0.01
ARS score	76.4 (13.0)	21	0.82	< 0.01
ARS—Novelty seeking	24.9 (5.4)	7	0.71	< 0.01
ARS—Emotional regulation	30.3 (5.4)	9	0.43	**0.25**
ARS—Positive future orientation	21.1 (5.7)	5	0.95	< 0.01

^*^Shapiro–Wilk W-test for normality.

*SD* standard deviation, *IPFI* Individual Protective Factors Index Questionnaire, *ARS* Adolescent Resilience Scale.

^Obtained by dividing the total score by the number of items

### Data analysis

We used STATA^®^ version 15 for data analysis and assumed a valid significance level of 0.05 for two-tailed hypothesis tests. We performed exploratory analyses to detect possible outliers and non-normal distributions utilizing the Shapiro–Wilk’s W-test for normality. We evaluated internal consistency of the resilience scales using Cronbach’s alpha and evaluated concordance and correlation between the scales and subscales utilizing Lin’s and Spearman’s coefficients, respectively. When possible, we used Bonferroni adjustment for multiple comparisons. There were no missing data on the resilience scales or demographics, except for 9 students who decided not to report whether they had failed academic years.

To make the results comparable between scales, we used standardized and ranked resilience scores. Standardizing a non-normal distribution does not make it into a normal distribution but allows for comparison between continuous scales, and it is a familiar method to many researchers and clinicians; the comparison of ranked scores is an alternative for comparing non-normal distributions, the procedure assigns a rank (position in the group) to every score, and the comparison is then done between ranks in different scales. Standardized scores were compared to look for individuals who scored under -2 standard deviations (SD) in both scales (as those would be at the largest risk), and ranked scores were compared using quartiles (looking for quartile-concordant scores among the two scales). Additionally, the Bland–Altman's limits-of-agreement procedure was used to verify the level of disagreement between ranked scores. This procedure averages the ranks obtained by every individual in both scales and compares that average with the difference between both ranks; in short, a high level of concordance would show a difference close to zero throughout the whole rank distribution.

## Results

Of the 448 students attending sixth grade in the three schools, 325 (41.5% female) participated in the study (72.5% response rate). Mean age was 12.1 years (SD: 1.04). One fifth of the respondents considered their family’s economic resources to be “insufficient” to their needs, and 8% reported having experienced hunger at least once in the past year, due to lack of resources. The students also reported high rates of failed academic years (32.3%, 102 out of 316, 9 missing data in this variable), frequent school absenteeism (17.5%), lack of health insurance despite access to universal health care (13.5%), overcrowding in housing (4 or more people in a single room, 12%) and child labor (12%). Half of the adolescents had changed their place of residence three or more times in their lifetime (IQR: 1–5), which in Colombia is considered another indicator of lower family SES and greater stressors.

**Fig. 1:**
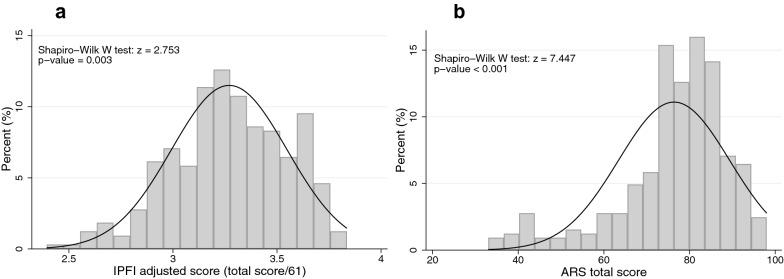
Resilience Levels in the Individual Protective Factors Index Questionnaire (IPFI) and the Adolescent Resilience Scale (ARS)

Standardized mean total scores of both scales were similar, both deviating from normality (p-value in the Shapiro–Wilk W-test for both scales < 0.01) towards the lower range of the distribution (see Fig. 1). The average IPFI adjusted score was 3.3 with a 0.3 standard deviation (SD) out of the possible scores (1–4) [equivalent to a total score of 201.3 out of possible scores 61–244], and the average ARS total score was 76.4 (SD 13.0) out of the possible scores (21–105). The internal consistency of the global scores of the scales was good, with a Cronbach alpha of 0.87 for the IPFI and 0.82 for the ARS. There was greater variability in the Cronbach alpha for the subscales of both instruments (Table 1).

**Fig. 2:**
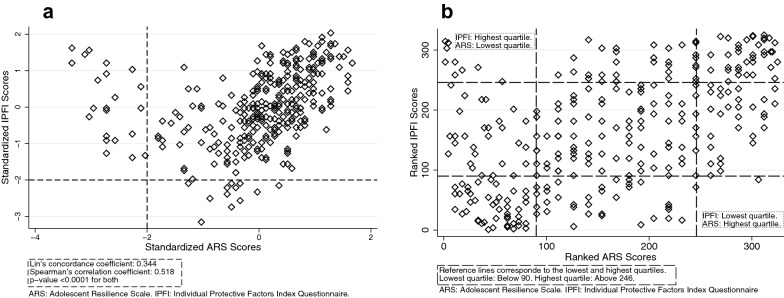
Concordance Between the Individual Protective Factors Index Questionnaire (IPFI) and the Adolescent Resilience Scale (ARS). Panel **A** (left) shows a scatter plot of standardized scores with a reference line on -2 standard deviations. Panel **B** (right) shows a scatter plot of ranked scores with reference lines on the lowest and highest quartiles in each scale

**Fig. 3:**
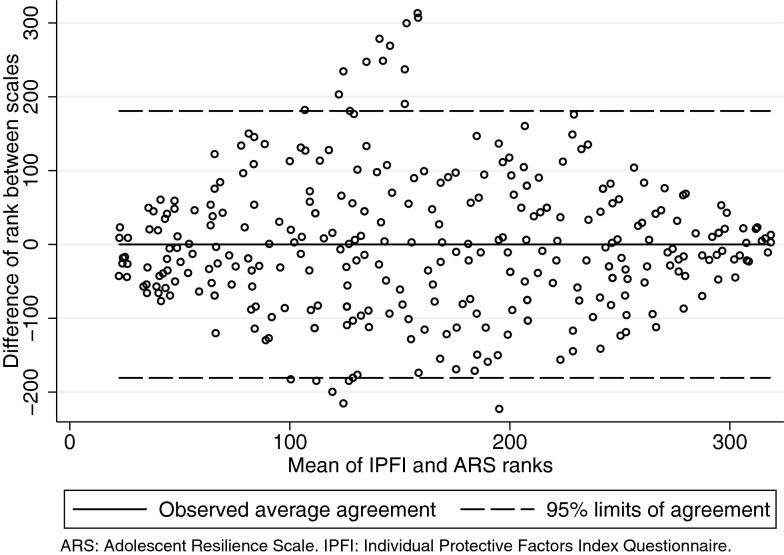
Bland–Altman’s analysis of limits of agreement between ranked scores of the Individual Protective Factors Index Questionnaire (IPFI) and the Adolescent Resilience Scale (ARS)

Lin’s concordance between the scales was fair (0.34) and Spearman’s was moderate (0.52) with a p-value < 0.0001 for both (Fig. 2). Thirty-four adolescents (10.5% of the sample) had a score lower than -2 SD in one of the two instruments but not in the other (22 for the ARS and 12 for the IPFI). We expected to have some individuals with low scores in both scales, but there are none (left-bottom quadrant of the distribution in Fig. 2a). Resilience scores ranked below 90 correspond to the lowest quartile of the distribution, and scores ranked above 246 correspond to the highest quartile. Out of the 86 adolescents in the lowest quartile of the IPFI, 47 were also in the lowest quartile of the ARS (left-bottom quadrant of the distribution in Fig. 2b). Out of the 81 adolescents in the highest quartile of the IPFI, 38 were also in the highest quartile of the ARS (right-top quadrant in Fig. 2b). However, 1 out of the 86 adolescents in the lowest quartile of the IPFI had a score in the highest quartile of the ARS (right-bottom quadrant of the distribution in Fig. 2b), and 9 out of the 81 adolescents with scores in the highest quartile of the IPFI had scores in the lowest quartile of the ARS (left-top quadrant in Fig. 2b). Overall, out of the 325 adolescents, only 144 scored in the same quartile in both resilience scales (44.31%). Bland–Altman’s analysis confirmed that some scores have higher than expected differences between ranks in both scales, approximately half of the sample has a difference in ranks between scales larger than 50 positions (Fig. 3).

Among subscales of the two instruments (Table 2), the strongest correlation was found between the self-control subscale of the IPFI and the emotional regulation subscale of the ARS, with a Spearman correlation coefficient of 0.44 (p-value < 0.0001). Notably, subscales of both instruments had a statistically significant correlation with most subscales in the other instrument, except for the novelty seeking subscale of the ARS which only showed a significant correlation with two IPFI subscales.

**Table 2:** Spearman’s Correlation Coefficients Between the IPFI and ARS Subscale Scores (N = 325)

IPFI-A												
0.356***	IPFI-B											
0.391***	0.307***	IPFI-C										
0.385***	0.409***	0.378***	IPFI-D									
0.390***	0.403***	0.409***	0.463***	IPFI-E								
0.193*	0.187	0.211**	0.353***	0.321***	IPFI-F							
0.288***	0.306***	0.319***	0.350***	0.253***	0.042	IPFI-G						
0.355***	0.292***	0.332***	0.420***	0.361***	0.244***	0.235**	IPFI-H					
0.275***	0.316***	0.428***	0.568***	0.408***	0.329***	0.315***	0.437***	IPFI-I				
0.357***	0.391***	0.454***	0.548***	0.430***	0.151	0.388***	0.312***	0.395***	IPFI-J			
0.163	0.177	0.213**	0.140	0.156	0.055	0.175	0.164	0.179	0.195*	ARS-A		
0.275***	0.167	0.258***	0.313***	0.444***	0.288***	0.078	0.276***	0.254***	0.157	0.137	ARS-B	
0.295***	0.309***	0.276***	0.315***	0.336***	0.141	0.257***	0.275***	0.234**	0.228**	0.544***	0.358***	ARS-C

^*^p-value < 0.05. **p-value < 0.01. ***p-value < 0.001. P-value with Bonferroni correction for multiple comparisons.

*IPFI-A* Social bonding—School, *IPFI-B* Social bonding—Family, *IPFI-C* Social bonding—Prosocial norms, *IPFI-D* Personal competencies – Self-concept, *IPFI-E*: Personal competencies – Self-control, *IPFI-F* Personal competencies – Self-efficacy, *IPFI-G* Personal competencies—Positive outlook, *IPFI-H* Social competencies—Assertiveness, *IPFI-*I Social competencies—Confidence, *IPFI-J* Social competencies—Cooperation, *ARS-A* Novelty seeking, *ARS-B* Emotional regulation, *ARS-C* Positive future orientation.

## Discussion

This study compared two measurement scales that evaluate intrapersonal aspects of psychological resilience in a sample of sixth-grade students from a low socio-economic, urban area in the capital city of Colombia. The self-reported socioeconomic indicators point to an overall disadvantaged population as one out of every five students reported either having insufficient family economic resources, engaging in child labor, experiencing overcrowded housing conditions or hunger due to economic problems. We found that overall concordance between the two scales was fair to poor, most strikingly among those identified as having “low” resilience levels. The distribution of resilience for both scales was negatively skewed (left tail), with a group of 34 students with a low resilience score in one of the two scales (standardized score below -2 SD in either scale, but not in both). Only 47 out of 86 students in the lowest quartile of the IPFI distribution were found to be in the same quartile of the ARS distribution, and 10 students had completely discordant scores (ranked in the lowest quartile of one scale and in the highest quartile of the other). Considering that the scales intend to measure the same construct, and both operationalize it using internal protective factors, we expected to find a greater correlation.

This finding may be explained by conceptual differences in the construct of resilience in both scales. The IPFI measures social bonding in three areas of life, four types of personal competencies (self-concept, self-control, self-efficacy, and positive outlook) and three types of social competencies (assertiveness, confidence, and cooperation); in contrast, the ARS evaluates three factors that could resemble the personal competencies of the IPFI (novelty seeking, emotional regulation and positive future orientation). Surprisingly, the two closest subscales, which refer to positive future orientation, did not have a clinically significant correlation between them (Spearman’s coefficient: 0.257, p < 0.001). The largest correlation found to be statistically significant, which was moderate (Spearman’s coefficient: 0.444, p < 0.001), was between the emotional regulation factor of the ARS and the self-control dimension of the IPFI. These subscales have similar items, such as “I have difficulty in controlling my anger” (ARS) and “I get mad easily” (IPFI) or “My behavior varies with my daily moods” (ARS) and “I do whatever I feel like doing” (IPFI) [19, 20]. Both subscales indicate the ability to control emotional changes, although the items in the ARS are more general, and items in the IPFI refer more to controlling negative emotions and aggressiveness. Many of the dimensions measured with the subscales of these two instruments correspond to what Pangallo et al. identify as the internal resources of the resilience construct: “adaptability, self-efficacy, active coping, positive emotions, mastery and hardiness” [16]. A new direction in research could be the use of multiple scales in studies that intend to capture all of those aspects of resilience; using a single scale could be misleading in identifying people who would benefit from interventions. Also, researchers could consider the possibility of using structural equation modeling with these resilience subscales to assess the correlation of the latent resilience variable between instruments, an approach that would correct some of the issues presented here.

It could be that our findings are a particular characteristic of this sample, but the distribution of data in this study is comparable to that reported in other studies of adolescents using the same scales. We did not find studies in Colombian adolescents using the IPFI, but studies in other populations of youth had similar results: Lussier et al. measured the levels of resilience in 1273 adolescents aged 12–19 years in Montreal (Quebec, Canada) and obtained an adjusted IPFI score of 3.15 (SD 0.35) [22]; Carlson evaluated 118 adolescents (10–15 years of age) in a public school in an urban area of the U.S. that serves students with socioeconomic risks, finding an average of 58.11 (SD 7.16, equivalent to an adjusted score of 3.22) for the social bonding subscale, of 80.1 (SD 8.82, equivalent to an adjusted score of 3.20) for the personal competencies subscale and of 57.34 (SD 7.59, equivalent to an adjusted score of 3.19) for the social competencies subscale [23]; finally, a study by Conners-Burrow et al. used only the self-control dimension of the IPFI, reporting 18.2 (SD 3.8, equivalent to an adjusted score of 3.03) in 60 children and adolescents (ages 7–11 years) in urban and rural areas of Arkansas (United States) [24]. Results in our study (Table 1) were similar in this scale with an adjusted score of 3.3 (SD 0.3), which would be equivalent to a total score of 201.3. As for the ARS, there are two Colombian studies with similar scores: Restrepo-Restrepo, Vinaccia and Quiceno reported a total score on the scale of 71.42 (SD 7.95) in 10–12-year-old students at a school in the city of Medellín (n = 36) [25], and Quiceno et al. presented an adjusted ARS score of 3.90 (SD 0.38) in slightly older adolescents (12–16 years) attending private schools in Bogotá (n = 686) [21]. Results in our study (Table 1) were remarkably similar with a total score of 76.4 (SD 13.0), which would be equivalent to an adjusted score of 3.64. It is clear from these studies that authors often use different ways of reporting data on the same instruments, making it difficult to interpret and compare date between studies. Even so, the similarity of scores in domestic and foreign samples is notable, given the different social economic and cultural backgrounds of the populations in each study.

The main limitations of our results originate in the very nature of the question at study and the absence of an operationalized construct or gold standard for evaluating resilience. The different interpretations of the construct contribute to the complexity of its conceptual mapping, as has been pointed out by others [26, 27]. Our study findings highlight that measuring levels of resilience requires a broader approach. For example, Ghimbulut and Opre used mixed methods to develop a measurement instrument that was relevant to their environment in Romania [28]. However, while more individualized approaches may increase local relevance, they can make interpretability of results even more difficult across studies. If the construct of resilience is to have value for prevention and health promotion, one would hope that scales would at least have some level of concordance with who is identified as at risk by each scale. In the absence of a more operationalized resilience construct, studies that include resilience scales should consider including a more in depth understanding of what aspects of the construct are being measured, and ideally, how they relate to meaningful variables in the population at study (for example, well-being or risk of illness, etc.). This strategy could aid in finding consensus on what aspects of the resilience construct are reliably measurable and assist in interpreting resilience scale scores when implementing mental health promoting interventions.

## Data Availability

Data available upon reasonable request from the corresponding author (ACCA): Individual level deidentified patient data, statistical code. ACCA confirms that he had full access to all the data in the study and takes responsibility for the integrity of the data and the accuracy of the data analysis.

## Abbreviations

ARS: Adolescent Resilience Scale
IPFI: Individual Protective Factors Index Questionnaire
IQR: Interquartile range
SD: Standard deviation
